# Lignans From *Forsythia x Intermedia* Leaves and Flowers Attenuate the Pro-inflammatory Function of Leukocytes and Their Interaction With Endothelial Cells

**DOI:** 10.3389/fphar.2018.00401

**Published:** 2018-04-24

**Authors:** Barbara Michalak, Agnieszka Filipek, Piotr Chomicki, Małgorzata Pyza, Marta Woźniak, Barbara Żyżyńska-Granica, Jakub P. Piwowarski, Agnieszka Kicel, Monika A. Olszewska, Anna K. Kiss

**Affiliations:** ^1^Department of Pharmacognosy and Molecular Basis of Phytotherapy, Medical University of Warsaw, Warsaw, Poland; ^2^Department of Pharmacodynamics, Faculty of Pharmacy, Centre for Preclinical Research and Technology, Medical University of Warsaw, Warsaw, Poland; ^3^Department of Pharmacognosy, Faculty of Pharmacy, Medical University of Lodz, Lodz, Poland

**Keywords:** *Forsythia x intermedia*, Oleaceae, lignans, enterolactone, pro-inflammatory mediators, neutrophils, macrophages, endothelial cells

## Abstract

**Aim of the study:** Taking into account that overactivated leukocytes are an important factor in the development of many chronic diseases, we investigated the activity of phytochemically characterized (HPLC-DAD-MS^n^) extracts from forsythia leaves and flowers on the pro- and anti-inflammatory functions of leukocytes (effects on IL-1β, IL-8, TNF-α, and TGFβ release) and their adherence to endothelial cells. Using bio-guided fractionation, we isolated the active compounds and determined their biological activity, and we included the positive control quercetin.

**Methods:** The effect on IL-1β, TNF-α, IL-8, and TGF-α production by leukocytes was measured by enzyme-linked immunosorbent assay (ELISA). The surface expression of adhesion molecules was analyzed with flow cytometry, and the neutrophil attachment to the endothelial cells was assessed fluorimetrically. The effects on p38MAPK, ERK1/2 and JNK phosphorylation were determined using western blots.

**Results:** Leaf extracts had the effect of decreasing TNF-α production in neutrophils and monocyte/macrophage cells. The bio-guided fractionation led to the isolation of the following lignan aglycones: (+)-pinoresinol, (+)-epipinoresinol, (−)-matairesinol, (+)-phillygenin, and (−)-arctigenin. Only phillygenin was able to stimulate the anti-inflammatory function of macrophages by inducing TGF-β release and IL-10 receptor surface expression. Arctigenin, phillygenin, and a metabolite produced by the gut microbiota, enterolactone, decreased TNF-α and IL-1β production and neutrophil adhesion to endothelial cells, probably by attenuating the p38 and ERK kinase pathways.

**Conclusion:** *Forsythia x intermedia* is a valuable source of active lignans, which may be potential candidates for treating inflammatory diseases that are associated with the excessive production of cytokines such as TNF-α and IL-1β.

## Introduction

The genus *Forsythia* Vahl (Oleaceae) comprises between 10 and 15 species, mostly native to Asia, and one native to southern Europe (Zhang et al., 2012). Forsythia fruits, which are usually obtained from *F. suspensa* (Thumb) Vahl and *F. viridissima* Lindley, are known in Asia as diuretic, hypotensive, anti-allergic, anti-inflammatory, antipyretic, anti-infective, and antidote agents. Moreover, the plant material is listed by the Chinese, Japanese, and Korean Pharmacopoeias (Tokar, 2002; Evans, 2009; Zhang et al., 2012). In Europe, aside from one native taxon (*F. europaea* Degen *et* Bald), the species that are present (*F. x intermedia, F. suspensa* and *F. viridissima*) are naturalized and cultivated as decorative shrubs (Tokar, 2002; Kicel et al., 2015). In temperate climates, these plants do not form fruits and, in Europe, more attention is paid to the leaves and flowers as a source of valuable compounds such as lignans: phillygenin-*O*-glucoside (phyllyrin), arctigenin-*O*-glucoside (arctiin), matairesinol-*O*-glucoside (matairesinoside), pinoresinol-*O*-glucoside; phenylethanoids: forsythoside A and verbascoside/acteoside; and flavonoids: quercetin-*O*-rhamnoglucoside (rutin) (Kitagawa et al., 1988; Rahman et al., 1990; Tokar and Klimek, 2004a; Kicel et al., 2015). These compounds have potential pharmacological activity and anti-inflammatory properties.

Inflammation is a consequential host defense response, and acute inflammation involves neutrophils, basophils, eosinophils, and mononuclear cells (monocytes/macrophages). The inflammation process is beneficial and stimulates the healing process. In contrast, persistent inflammation lasting months or even years is an underlying cause of many chronic diseases, such as atherosclerosis, diabetes mellitus, obesity, osteoporosis, rheumatoid arthritis, inflammatory bowel disease, asthma, and others (Baetta and Corsini, 2010; Laveti et al., 2013; Steyers and Miller, 2014; Al-Soudi et al., 2017). Neutrophils, also known as polymorphonuclear cells (PMN), are released into the circulation and are the most abundant type of leukocytes. Once released, neutrophils begin to first signs of inflammation, and through a series of events, they undergo transmigration through the vessel wall toward the inflamed tissue. The attachment of neutrophils to the vascular endothelium is the first step and is essential for their accumulation. Adhesion molecules, such as β2 integrin CD11a/CD18 (LFA-1) and CD11b/CD18 (Mac-1), are responsible for the firm adhesion and transmigration through the endothelium to inflamed tissues. After infiltration of the inflamed site, they generate oxidative burst and release proteases [elastase, matrix metalloproteinases (MMPs)], chemokines and cytokines (interleukins IL-8, IL-1β; TNF-α). During chronic inflammation, neutrophils are activated by a variety of stimuli, which increases their pro-inflammatory attributes. Moreover, several cytokines and proteases expressed by neutrophils promote a systemic inflammation, modulate endothelial permeability and affect the endothelial and smooth muscle cell responses (Witko-Sarsat et al., 2000; Baetta and Corsini, 2010; Tintinger et al., 2013). Endothelial cells (ECs) undergo inflammatory stimulation resulting in increased expression of adhesion molecules such as selectins (E and P), vascular cell adhesion molecule-1 (VCAM-1), and intercellular adhesion molecule-1 (ICAM-1), which promote the adherence of the inflammatory cells: monocytes, neutrophils, lymphocytes, and macrophages. If vascular inflammation remains unresolved, it may lead to vascular diseases such as atherosclerosis, abdominal aortic aneurysm, varicose veins, and hypertension (Sprague and Khalil, 2009). Tumor necrosis factor (TNF-α) is one of the factors that induce cell adhesion molecule expression in ECs, and it is associated with the activation of multiple signal transduction pathways (Steyers and Miller, 2014). Activated ECs secrete chemoattractant mediators that promote mononuclear cell recruitment into the vascular wall. Monocytes differentiate into macrophages, which become foam cells after ox-LDL uptake and then release a variety of pro-inflammatory cytokines, such as soluble CD40 ligand, IL-1, IL-3, IL-8, IL-18, and TNF-α (Golia et al., 2014). The most important mechanism to locally eliminate inflammatory cells is phagocytosis by recruited monocyte-derived macrophages. Monocytes recognize cells undergoing apoptosis and efficiently and rapidly remove them. This process not only eliminates inflammatory cells but also contributes to the resolution of inflammation via the production of TGF-β and IL-10 by macrophages (Zhang and Mosser, 2008).

The aim of the present study was to investigate the effects of phytochemically characterized (HPLC-DAD-MS^n^) extracts from forsythia leaves and flowers on the pro- and anti-inflammatory functions of leukocytes (effects on IL-1β, IL-8, TNF-α, and TGFβ release) and their adherence to endothelial cells. To isolate the active compounds and determine their biological activity and usefulness as potential lead compounds, we performed bio-guided fractionation. We also focused our attention on possible metabolites produced by gut microbiota from active and/or precursor compounds. Finally, we focused on the molecular mechanisms of active compounds that regulate neutrophil inflammation.

## Materials and methods

### Chemicals and general experimental procedures

LPS (from *Escherichia coli* 0111:B4), HEPES buffer, L-glutamine, Hank's balanced salt solution (HBSS), RPMI 1,640 medium, and Cell Dissociation Solution (non-enzymatic) were purchased from Sigma-Aldrich Chemie GmbH (Steinheim, Germany). Fetal bovine serum (FBS) and phosphate-buffered saline (PBS) were purchased from Gibco (Grand Island NY, USA). Ficoll Hypaque gradient (LSM 1077) and penicillin–streptomycin were obtained from PAA Laboratories, GmbH (Pasching, Austria). Human Quantikine ELISA Kits were purchased from R&D System (Minneapolis, USA). Anti-human CD11b (conjugated with phycoerythrin, PE) was purchased from eBioscience (San Diego, CA). Anti-human CD11a (conjugated with fluorescein isothiocyanate, FITC), anti-human CD54 (ICAM) (conjugated with allophycocyanin, APC), anti-Human CD62E (conjugated with phycoerythrin, PE) and anti-human IL-10 (conjugated with phycoerythrin, PE) were purchased from BD Pharmingen. Anti- pp38 (#9211), ppJNK (#9251), pJNK (#9252), pERK1/2 (#9102), NF-κB (p65) (#8242S), and β-actin (#4967) antibodies were purchased from Cell Signaling Technology (USA). Anti- ppERK1/2 (#V8031) was purchased from Promega (USA) and anti-p38 (#SC-535) from Santa Cruz Biotechnology (USA).

Accutase™ Cell Detachment Solution and propidium iodide were purchased from BD Biosciences. EGM-2 BulletKit was purchased from Lonza (Basel, Switzerland). The HUVEC line was purchased from Invitrogen (Carlsbad, USA). Calcein-AM was obtained from MoBiTec GmbH (Göttingen, Germany). Enterolactone was purchased from Sigma-Aldrich Chemie GmbH (Steinheim, Germany). Quercetin was purchased from Carl Roth (Karlsruhe, Germany).

NMR spectra were recorded on a Varian VNMRS 300 MHz spectrometer in CDCl_3_ (aglycones) or DMSO-*d*_6_ (glycosides). Preparative HPLC was performed with a Shimadzu LC-20AP instrument (Japan) using a KinetexXB-C18 column (Phenomenex, USA, particle size 5.0 μm, 150 × 21.2 mm) at a flow rate of 20.0 mL /min. TLC was performed on Merck silica gel 60 F 254 (0.25 mm) with dichloromethane/methanol/formic acid/water (80:25:1.5:4; v/v/v/v). Chromatograms were visualized by spraying with sulfuric acid (5% in methanol) followed by heating at 105°C for 10 min. All solvents used for chromatography were of gradient grade. HPLC-DAD-MS^n^ analysis was performed on a UHPLC-3000 RS system (Dionex, Germany) with DAD detection and an AmaZon SL ion trap mass spectrometer with ESI interface (Bruker Daltonik GmbH, Germany). Separation was performed on a Zorbax SB-C18 column (150 × 2.1 mm, 1.9 μm) (Agilent, USA). The mobile phase consisted of 0.1% HCOOH in water (A) and 0.1% HCOOH in MeCN (B) using the following gradients: 0–60 min, 5–40% B. The LC eluate was introduced into the ESI interface without splitting, and compounds were analyzed in the negative ion modes with the following settings: nebulizer pressure of 40 psi; drying gas flow rate of 9 L/min; nitrogen gas temperature of 300°C; and a capillary voltage of 4.5 kV. The mass scan ranged from 100 to 2,200 m/z. UV spectra were recorded in the range of 200–400 nm.

The absorbance and fluorescence were measured using a BioTek microplate reader (Highland Park, USA). Flow cytometry was performed using a BD FACSCalibur apparatus (BD Biosciences, San Jose, CA, USA).

### Plant material

The flowers and leaves of *Forsythia x intermedia* Zabel were collected in April and June 2016, respectively, in the Arboretum, Forestry Experimental Station of Warsaw University of Life Sciences in Rogow, Poland (51°49′ N; 19°53′E). The identity of the plant material was confirmed by Piotr Banaszczak, Head of the Arboretum, Forestry Experimental Station of Warsaw University of Life Sciences. A voucher specimen (FINT-F-2013) has been deposited in the herbarium of the Department of Pharmacognosy, Medical University of Lodz, Poland.

### Extract preparation, phytochemical characterization, and isolation of active compounds

The air-dried flowers (180 g) were crushed and extracted six times with 75% methanol (1: 20) at 70°C for 2 h each time. The methanol from combined extracts was evaporated under reduced pressure, and the aqueous residue was lyophilized to yield 105 g of extract (FFE).

The air-dried leaves (400 g) were crushed and extracted six times with 75% methanol (1: 20) at 70°C for 2 h each time. The methanol from combined extracts was evaporated under reduced pressure, and the aqueous residue was lyophilized to yield 188 g of extract (FLE).

The extracts were characterized by using an HPLC-DAD-MS/MS method. The presence of substances in extracts was confirmed by comparing the retention time and spectra (UV, MS, MS/MS) with standards and/or published data.

The crude FFE and FLE extracts were subjected to Diaion HP-20 (Supelco) column chromatography (30 × 6 cm) and eluted with an H_2_O-MeOH gradient (80: 20 → 0: 100) of 4 steps, 1,250 mL each, to obtain 20 fractions of 250 mL, which were pooled into 4 main fractions (FF1–FF4 and FL1–FL4) based on their TLC and HPLC profiles. The activity of the fractions on the inhibition of IL-8 and TNF-α was tested at a concentration of 10 μg/mL. Fraction FF4 and FL4 inhibited IL-8 production (48.7 ± 3.0% and 86.1 ± 7.8% of release compared with the stimulated control 100%, respectively) and TNF-α production (4.7 ± 0.2% and 34.5 ± 14.9% of release compared with the stimulated control 100%, respectively). Fractions FF4 and FL4 were subjected to further separation. Fraction FF4 (6.8 g) was separated again by chromatography on a Sephadex LH-20 (Pharmacia) column (20 × 5 cm) with H_2_O-MeOH (70:30) to obtain 20 fractions of 50 mL each, which were pooled into 4 main fractions (FFA_1-FFA_4) based on their TLC and HPLC profiles. From fractions FFA_2 (1.3 g) **(**+**)-epipinoresinol-4**′**-*O*-glucoside** (2 mg; RT = 8.6–8.9 min), **(**−**)-matairesinol-4**′**-*O*-glucoside** (13 mg; RT = 9.9–10.2 min), **(**+**)-phillygenin-4-*O*-glucoside** (160 mg; RT = 11.3–11.6 min), **(**−**) -arctigenin-4**′**-*O*-glucoside** (292 mg; RT = 11.8–12.5 min) and (−)-**arctigenin** (31 mg; RT = 19.4–19.9) were isolated using preparative HPLC with a 0.1% HCOOH in H_2_O (A)-0.1% HCOOH in MeCN (B) gradient (85: 15 → 0: 100) in 60 min. From fraction FFA_3 (0.7 g), **(**+**)-pinoresinol** (3 mg; RT = 13.7–14.1 min), **(**+**)-epipinoresinol** (3 mg; RT = 15.1–15.6 min), and **(**−**)-matairesinol** (6 mg; RT = 16.0–16.5 min) were isolated using preparative HPLC using the same conditions as for fraction FFA_2.

Fraction FL4 (19 g) was separated on a Sephadex LH-20 (Pharmacia) column (20 × 5 cm) with H2O-MeOH (70:30) to obtain 15 fractions of 50 mL each, which were pooled into 4 main fractions (FLA_1–FLA_4) based on their TLC and HPLC profiles. Fraction FLA_3 (2.4 g) was separated on a Sephadex LH-20 (Pharmacia) column (85 × 2.5 cm) with MeOH to obtain 35 fractions of 10 mL each, which were pooled into 6 main fractions (FLA_3.1–FLA_3.6) based on their TLC profiles. From fraction FLA_3.4 (0.78 g), **(**+**)-forsythoside A** (62 mg; RT = 6.3–6.6 min), **acteoside** (45 mg; RT = 6.8–7.1 min), **epipinoresinol-4**′**-*O*-glucoside** (16 mg; RT = 8.6–8.9 min), **(**+**)-phillygenin-4-*O*-glucoside** (35 mg; RT = 11.3–11.6 min), **(**+**)-epipinoresinol** (5 mg; RT = 15.1–15.6 min), and **(**−**)-matairesinol** (3 mg; RT = 16.0–16.5 min) were isolated using preparative HPLC using the same condition as for fraction FFA_2.

From fraction FLA_4 (1.7 g), additionally **(**+**)-phillygenin** (20 mg; RT = 18.7–19.3 min), and (−)-**arctigenin** (7 mg; RT = 19.4–19.9) were isolated by preparative HPLC using the same conditions as for fraction FFA_2.

### Production of enterolactone from (+)-phillygenin-4-*O*-glucoside and (−)-arctigenin-4′-*O*-glucoside by human gut microbiota

A human fecal sample was donated by a 30-year-old healthy male volunteer with no history of bowel diseases. The donor had not taken any antibiotics in the 6 months before feces collection. The study complied with the Helsinki Declaration. The consumption of lignan-containing products was reduced for 1 week before sample collection. Samples were processed within 30 min after defecation. The growth medium, brain heart infusion (BHI) (DIFCO, Detroit, MI, USA), was prepared according to the manufacturer's instructions. To establish anaerobic conditions, the BHI was boiled for 20 min and immediately cooled before the experiment. Fecal slurries (FS) were prepared by suspending human feces in BHI (1:10 w/v). Solutions of each compound (10 mg/mL) were prepared in deionized water and sterilized by filtration through 0.2-mm Ophtalsart hydrophilic syringe filters (Sartorius Stedim Biotech GmbH, Göttingen, Germany). Then, 1 mL of FS and 0.5 mL of compound solution were added to 8.5 mL of BHI. Subsequently, 0.5 mL of deionized water was added to the control blank sample. The batch cultures were incubated in sealed containers under anaerobic conditions using GENbox anaer sachets (bioMerieux, France) at 37°C. Compounds without FS were incubated in parallel. After 24 h, cultures were extracted with EtOAc (100 mL) and evaporated to dryness at 40°C. The evaporated fraction was dissolved in 1.0 mL of MeOH, subjected to an ultrasonic bath for 5 min and filtered through a 0.45-μm Chromafil polyester membrane (Macherey-Nagel, Duren, Germany). UHPLC-DAD-MSn analyses were performed using a UHPLC-3000 RS system (Dionex, Sunnyvale, CA, USA) with DAD detection and an AmaZon SL mass spectrometer with ESI interface (Bruker Daltonik GmbH, Bremen, Germany). The column was a Zorbax SB-C18 (150 mm × 2.1 mm × 1.9 μm) (Agilent, Santa Clara, CA, USA). The mobile phase consisted of 0.1% HCOOH in H2O (A) and 0.1% HCOOH in MeCN (B). The gradient was as follows: 0–5 min 0% B, 5–15 min 0–10% B, 15–25 min 10–20% B and 25–35 min 20–30% B, 35–45 min 30–50% B, 45–50 min 50–100% B, and 50–60 min 100% B. The column temperature was maintained at 25°C, and the flow rate was 0.200 mL/min. The LC eluate was introduced into the ESI interface without splitting, and compounds were analyzed in the negative and positive ion modes with the following settings: nebulizer pressure of 40 psi; drying gas flow rate of 9 L/min; nitrogen gas temperature of 300°C; and a capillary voltage of 4.5 kV. The mass scan ranged from 100 to 2,200 m/z. UV spectra were recorded in the range of 200–400 nm.

### Preparation of solutions of extracts and compounds for bioassay

Tested extracts were dissolved in DMSO (10 mg/mL). All test compounds and the positive control quercetin were dissolved in DMSO (10 mM stock solution) and then diluted with (Ca^2+^)-free HBSS and (Ca^2+^)-free PBS buffers at pH 7.4 or in RPMI 1,640 medium. The extracts were tested in a concentration range of 25–100 μg/mL. Compounds were tested at concentrations of 10–50 μM. The concentration of DMSO used (<0.1%) did not influence the assays.

### Isolation of human neutrophils and monocytes

Peripheral venous blood was obtain from healthy human donors (18–35 years old) in the Warsaw Blood Donation Centre. Donors were no smokers and received no pharmacotherapy. They were clinically recognized to be healthy and a routine laboratory test showed all values to be within the normal ranges. The study conformed to the principles of the Declaration of Helsinki.

Neutrophils were isolated by dextran sedimentation and centrifugation in a Ficoll Hypaque gradient and then re-suspended in an appropriate medium, such as (Ca^2+^)-free HBSS, (Ca^2+^)-free PBS at pH 7.4 or RPMI 1,640 medium.

Monocytes were isolated immediately after collection using a Ficoll Hypaque gradient as described by Zapolska-Downar et al. (2006). The mononuclear cell band was removed by aspiration, and cells were suspended in RPMI 1,640 medium with L-glutamine, HEPES, antibiotics and autologous serum (5%). To promote the adherence of monocytes/macrophages, the peripheral blood mononuclear cell suspension was placed in 12-well tissue culture plates (2 × 10^6^ per well) and incubated for 2 h at 37°C under humidified 5% CO_2_ air. After the incubation, non-adherent cells were removed, and adherent cells were cultivated in the same medium and conditions for 3 days. The medium and autologous serum were replaced every 2 days.

### Cell culture human umbilical vein endothelial cells (HUVECs)

HUVECs were purchased from Invitrogen Life Technologies. Cells were grown in EBM-2 basal medium supplemented with the EGM-2 SingleQuots kit. For all experiments, HUVECs were used at passage four. Cells were grown to confluence and then treated with extracts/compounds for 24 h in full EGM-2 medium.

### Cytotoxicity

Cytotoxicity (cell wall integrity) was determined by a flow cytometry using propidium iodide (PI) staining or by using LDH release assay kit (Roche Applied Science). After 24 h of incubation with extracts/compounds, the neutrophils or HUVECs were harvested and centrifuged (1,500 RPM; 10 min; 4°C), washed once with cold PBS and re-suspended in 400 μL of PBS. Five microliters of PI (50 μg/mL) solution was added to the cell suspensions. After 15 min of incubation at room temperature, cells were analyzed by cytometry, and 10,000 events were recorded per sample. Cells that displayed high permeability to PI were expressed as a percentage of PI(+) cells. After 24 h of incubation with extracts/compounds, with monocyte/ macrophage cells, the supernatants were harvested and the LDH level was measure according to manufacturer's instruction. The percentage of death cells was calculated based on the effect of Triton X-100 (100% of cytotoxicity).

### IL-8, IL-1β, and TNF-α production

Neutrophils (2 × 10^5^) were cultured in 24-well plates in RPMI 1,640 medium with 10% FBS, 10 mM HEPES, and 2 mM L-glutamine in the presence or absence of LPS (100 ng/mL) for 24 h at 37°C with 5% CO_2_ in the presence or absence of test extracts/compounds. After 24 h, the neutrophils were harvested and centrifuged (2,000 RPM; 10 min; 4°C). The amount of released cytokines was measured by enzyme-linked immunosorbent assay (ELISA) following the manufacturer's instructions (BD Biosciences, USA). The effects on IL-8, IL-1β, and TNF-α production were calculated by comparing the percentages of the released agents to the stimulated control, which lacked the test compounds.

### Western blotting

Neutrophils (4 × 10^6^) were suspended in RPMI 1,640 medium and incubated for 40 min at 37°C in the presence or absence of LPS (100 ng/mL) and in the presence or absence of the test compounds. They were then centrifuged at 1,500 RPM for 10 min at 4°C. Cells were lysed in ice-cold buffer containing PBS, 5 mM EDTA, 1% Triton X-100, phosphatase and protease inhibitors, and they were centrifuged at 8,000 RPM for 15 min at 4°C. The proteins were separated by 12% SDS-PAGE. Proteins were transferred to nitrocellulose filters and immunoblotted with rabbit anti-p38MAPK, anti-ERK1/2, anti-JNK, and anti-NF-κB-p65 at 1:2000 dilution and a rabbit anti-actin polyclonal antibody at 1:2000 dilution. Peroxidase-conjugated affinipure goat anti-rabbit antibody was used as a secondary antibody at a dilution of 1:10000. Finally, the blots were incubated with chemiluminescent substrate for detection of HRP (Thermo-Scientific, USA) for 10–15 min.

### CD11a/CD 18 and CD11b/CD18 adhesion molecule surface expression

The cell suspensions (1 × 10^6^) were preincubated with 100 μL of extracts/compounds for 30 min at 37°C, and LPS (1 μg/mL) was then added to the cells. After stimulation, the cells were marked with a monoclonal antibody against CD 11a and/or CD11b and analyzed by flow cytometry. The effect on the surface expression of adhesion molecules was evaluated based on a software-generated marker histogram M1 for LPS-stimulated cells.

### Expression of E-selectin and ICAM on the surfaces of endothelial cells

Human umbilical vein endothelial cells (HUVECs) were incubated with extracts/compounds for 24 h then followed by TNF-α treatment (10 ng/mL, 4 h). Cell monolayers were washed with PBS and suspended in 200 μL of accutase. After 10 min incubation at 37°C, the cells were harvested and centrifuged (1,500 RPM; 10 min; 4°C) and suspended in 300 μL of (Ca^2+^)-free PBS buffer. Cells were marked with anti-human CD62E and/ or CD54 and analyzed by flow cytometry. The effect on the surface expression of adhesion molecules was evaluated based on a software-generated marker histogram M1 for TNF-α stimulated cells.

### The attachment of neutrophils to endothelial cells

#### Neutrophils incubated with extracts

Neutrophils were incubated for 30 min at 37°C with or without extracts and then stimulated with LPS (100 ng/mL) for an additional 30 min. HUVECs were grown for 20 h, then treated with TNF-α (10 ng/mL, 4 h). Cell monolayers were washed and incubated for 30 min at 37°C with 200 μL of RPMI 1,640 medium and 100 μL of 1 mM calcein-AM labeled and pretreated neutrophils (1 × 10^6^).

#### Endothelial cells incubated with extracts

HUVECs were incubated with or without extracts for 20 h, which was followed by TNF-α treatment (10 ng/mL, 4 h). Cell monolayers were washed and incubated for 30 min at 37°C with 200 μL of RPMI 1,640 medium and 100 μL of 1 mM calcein-AM labeled LPS (100 ng/mL) stimulated neutrophils (1 × 10^6^).

#### Endothelial cells and neutrophils incubated with extracts/compounds

HUVECs were incubated with or without extracts/compounds for 20 h, followed by TNF-α treatment (10 ng/mL, 4 h). Cell monolayers were washed and incubated for 30 min at 37°C with 200 μL of RPMI 1,640 medium and 100 μL of 1 mM calcein-AM labeled neutrophils (1 × 10^6^). Previously, neutrophils were incubated for 30 min at 37°C with or without extracts/compounds and then stimulated with LPS (100 ng/mL) for an additional 30 min.

Next, the cell monolayers with attached neutrophils were washed, and the morphology of the HUVECs with attached neutrophils was assessed by using an Eclipse TS100F microscope (Nikon, USA) for visual light and fluorescence imaging. Cell monolayers with attached neutrophils were then lysed with 0.1% Triton X-100, and fluorescence was measured in a microplate reader at 485 nm excitation and 520 nm emission.

### Expression of IL-10 receptor on the surfaces of monocytes/macrophages

The expression of IL-10 receptor on the surfaces of monocytes/macrophage cells was determined by flow cytometry. Cells were incubated with LPS at a concentration of 100 ng/mL for 1 h, and they were then incubated with each extract/compound for 24 h. All solutions were added to the cells on the second day of incubation. Cells then were removed, centrifuged (13,000 RPM, 4°C, 1 min), suspended in PBS (100 μL), and incubated with the antibody for 20 min at 4°C. The mean fluorescence intensity in the gated cell population was measured (10,000 cells per sample) and analyzed by flow cytometry. The results were expressed as the percent of cells expressing IL-10 receptor in comparison to control cells stimulated by LPS.

### TNF-α and TGF-β production by monocytes/macrophages

Cells were cultured in 24-well plates in the presence or absence of LPS (100 ng/mL) and tested extracts/compounds for 24 h at 37°C with 5% CO_2_. After 24 h, cells were harvested and centrifuged (2,000 RPM; 10 min; 4°C). The amount of released cytokines was measured by ELISA following the manufacturer's instructions (BD Biosciences, USA). The effects on TNF-α and TGF-β production were calculated by comparing the percentages or pg/mL concentrations of the released agent to the control cells, which were stimulated but were not exposed to the test compounds.

### Statistical analysis

The results were expressed as the mean ± SEM of three independent experiments performed at least in duplicate. All analyses were performed using Statistica 9 software. The statistical significance of the differences between means was established by ANOVA with Dunnett's *post hoc* test. *P* values below 0.05 were considered statistically significant.

## Results

### Phytochemical characterization and comparison of *Forsythia x intermedia* flower and leaf extracts

**Figure 1:**
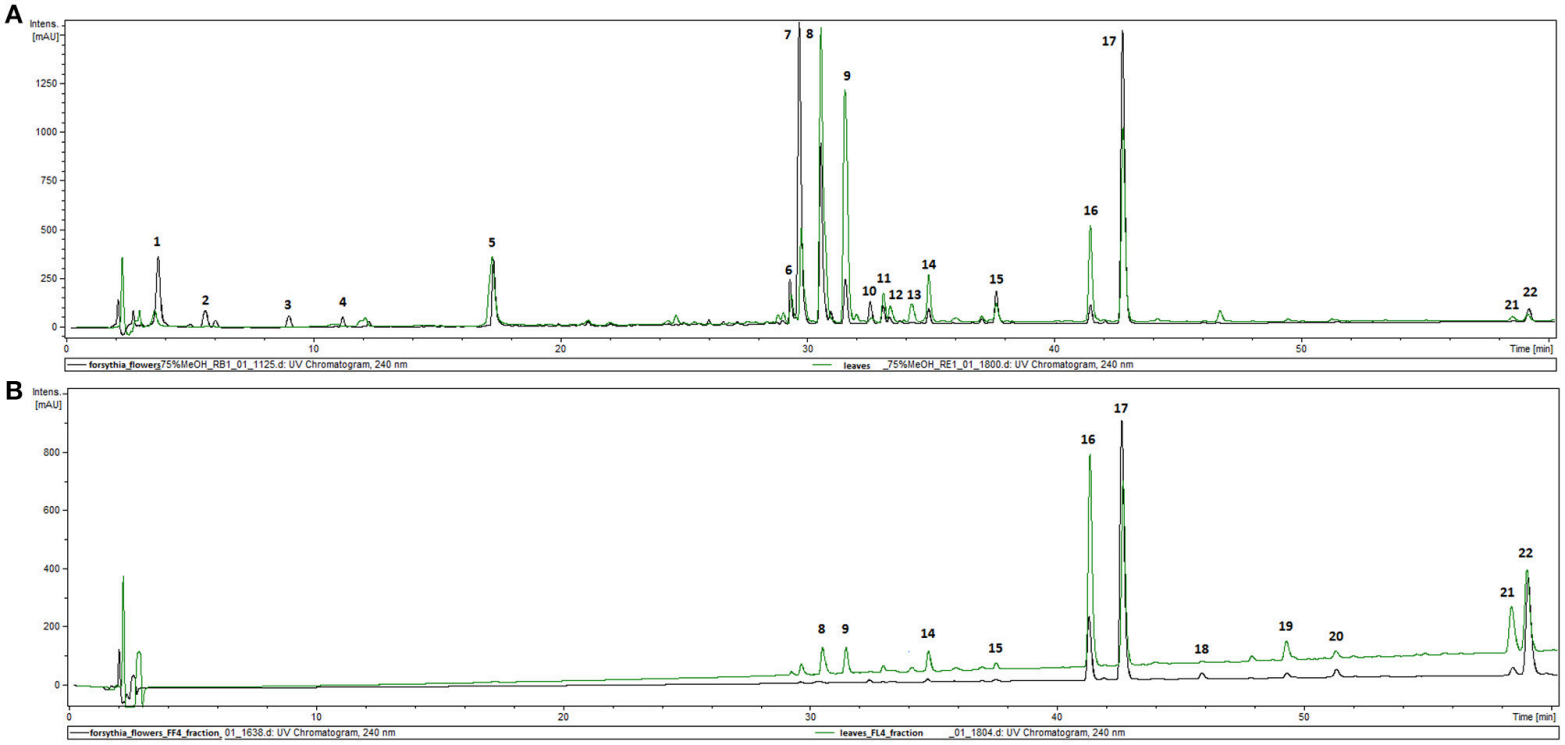
HPLC-DAD chromatograms of extracts (FFE and FLE) from F. x intermedia **(A)** and fractions FF4 and FL4 **(B)** recorded at 240 nm.

The phytochemical analysis of *Forsythia* x *intermedia* flower and leaf extracts (FFE and FLE) was performed by using the HPLC-DAD-MS/MS method, which allowed for the identification of 22 compounds (Table 1, Figure 1A) and confirmed the results of previous studies that showed that *Forsythia* x *intermedia* is a rich source of flavonoids, such as rutin (quercetin-3-*O*-rhamnoglucoside, Rt = 29.8 and *m*/*z* 609 [M-H]^−^), phenylethanoids: forsythoside A (Rt = 30.5 min and *m*/*z* 623 [M-H]^−^) and acteoside (Rt = 31.5 min and *m*/*z* 623 [M-H]^−^), and lignans from both dibenzylbutyrolactone: arctiin (arctigenin-4′-*O*-glucoside, Rt = 42.7 min and *m*/*z* 623 [M-H]^−^) and furofuran: phillyrin (phillygenin-4-*O*-glucoside, Rt = 41.1 min and *m*/*z* 623 [M-H]^−^) classes (Rahman et al., 1990; Tokar and Klimek, 2004a; Kicel et al., 2015). The phytochemical profiles of both extracts were similar, and only quantitative differences were observed (Figure 1A). Additionally, in the flower extract, some minor compounds such as cornoside (Rt = 3.8 min and *m*/*z* 315 [M-H]^−^) and adoxosidic acid (Rt = 11.2 min and *m*/*z* 375 [M-H]^−^) were identified (Endo and Hikino, 1984; Damtoft et al., 1994).

**Table 1:** Retention time, UV, and MS/MS data of the compounds present in flowers and leaf extracts from *F. intermedia* (FFE and FLE).

**No**	**Compounds**	**t_R_ [min]**	**UV [nm]**	**[M–H]^−^ (*m*/*z*)**	**MS^2^ ions**	**MS^3^ ions**	**References**
1.	Cornoside	3.8	230	361^*^	**315**	–	Endo and Hikino, 1984
2.	Unknown	5.6	250	405	**191**, 213	–	–
3.	Vanillic acid hexoside	9.0	212, 254, 290	659^**^	**329**, **167**	–	Dini et al., 2004
4.	adoxosidic acid	11.2	232	375	**213**	–	Damtoft et al., 1994
5.	Chlorogenic acid^a^	17.3	235, sh 296, 326	353	**191**, 179	–	Kicel et al., 2015
6.	Quercetin rhamnohexoside	29.2	255, 354	609	**301**	–	Kicel et al., 2015
7.	Quercetin-3-rhamnoglucoside^a^	29.8	256, 354	609	**301**	–	Kicel et al., 2015
8.	Forsythoside A	30.5	242, sh 290, 328	623	**461**, 477	**315**, 161, 135	Kicel et al., 2015
9.	Acteoside^a^	31.5	245, sh 292, 329	623	**461**	**315**, 135	Kicel et al., 2015
10.	Pinoresinol glucoside^a^	32.5	225, 277	519	**357**	342, 311, **151**, 136	Guo et al., 2007; Kicel et al., 2015
11.	Kaempferol rhamnohexoside	33.0	265, 342	593	**285**	–	Kicel et al., 2015
12.	Phenylpropanoid izomer	33.4	sh 285, 323	623	**461**, 477	**315**, 161, 135	–
13.	Epipinoresinol glucoside (I)	34.2	228, 278	565^*^	519, **357**	–	Guo et al., 2007
14.	Epipinoresinol glucoside (II)	34.8	228, 278	565^*^	519, **357**	–	Guo et al., 2007
15.	Matairesinol glucoside	37.6	222, 280	519	**357**	342, **313**, 298, 281, 209	Choi et al., 2003; Guo et al., 2007; Kicel et al., 2015
16.	Phillygenin glucoside	41.1	223, 278	579^*^	533, **371**	–	Guo et al., 2007; Kicel et al., 2015
17.	Arctigenin glucoside	42.7	230, 278	579^*^	533, **371**	–	Guo et al., 2007; Kicel et al., 2015
18.	Pinoresinol	45.8	225, 280	–	–	–	Guo et al., 2007;
19.	Epipinoresinol	49.2	223, 280	–	–	–	Guo et al., 2007;
20.	Matairesinol	51.0	220, 280	357	**342**, **313**, 298, 281, 209	–	Choi et al., 2003; Guo et al., 2007
21.	Phillygenin	58.0	220, 280	371	356	–	Guo et al., 2007;
22.	Arctigenin	58.6	222, 280	371	356, 295, 209	–	Choi et al., 2003

a confirmed by comparison with standard;

* [M-H+HCOOH]–;

** [2M-H]–. Bold values indicate most abundant fragmentation ion.

### Effect of *Forsythia x intermedia* flower and leaf extracts (FFE and FLE) on the pro-inflammatory function of LPS-stimulated neutrophils and monocytes/macrophages

**Figure 2:**
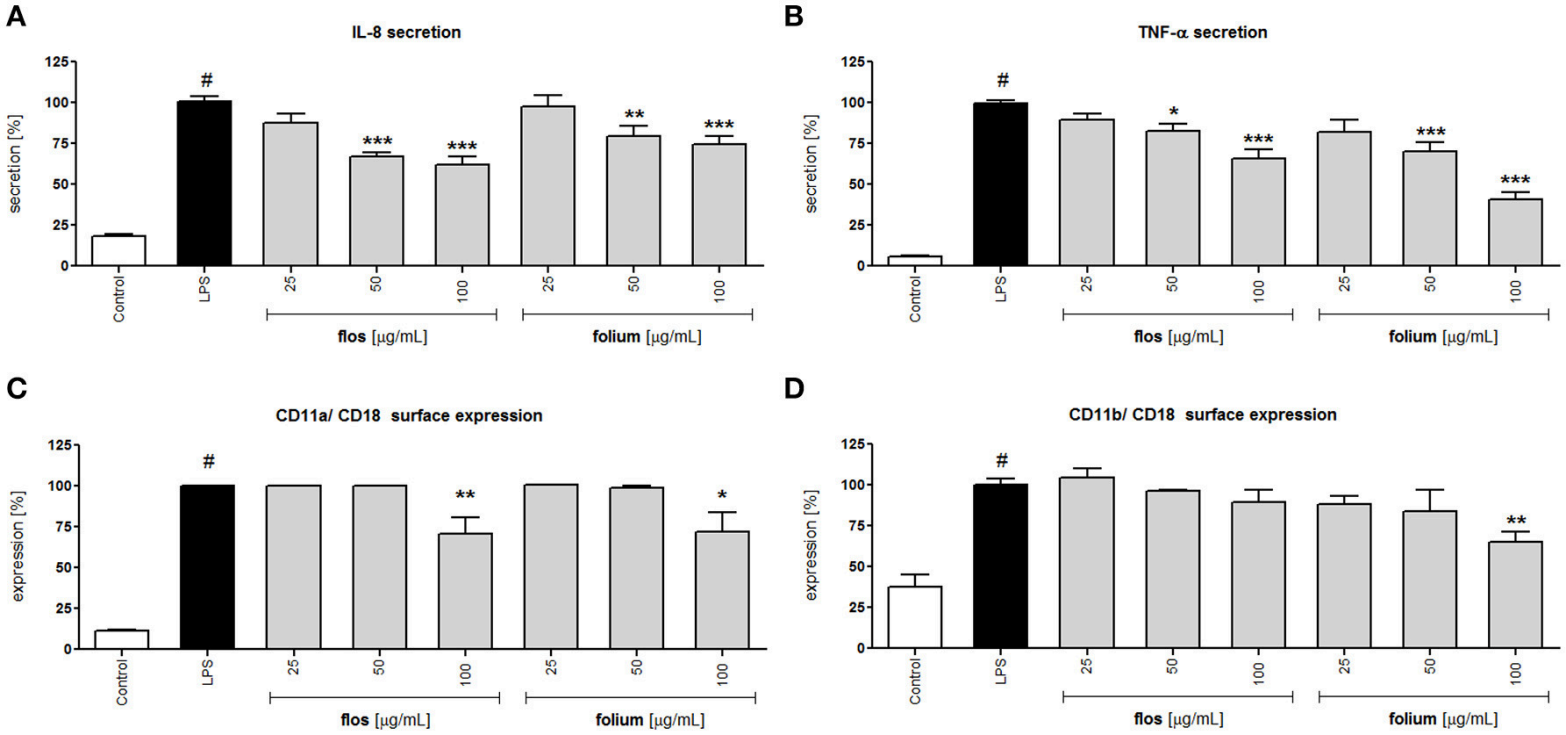
Effects of *F. x intermedia* flower and leaf extracts (FFE and FLE) at concentrations of 25–100 μg/mL on the pro-inflammatory function of neutrophils: **(A)** IL-8 secretion from stimulated neutrophils [%]; **(B)** TNF-α secretion from stimulated neutrophils [%]; **(C)** β2 integrin (CD11a/CD18) expression on the surface of stimulated neutrophils [%]; **(D)** β2 integrin (CD11b/CD18) expression on the surface of stimulated neutrophils [%]. Data were expressed as the mean ± SEM; at least three independent experiments were conducted, and they were assayed in duplicate. Experiments were performed using cells from different donors. Statistical significance ^#^*P* < 0.01 compared to the non-stimulated control; *P < 0.05, **P < 0.01, ***P < 0.001 decrease compared to stimulated control (LPS).

Activation of neutrophils by LPS resulted in greater surface expression of the adhesion molecule β2 integrin (CD11a/Cd18 and CD11b/CD18) and greater release of IL-8 and TNF-α compared to the untreated control (Figure 2). Incubation for 24 h of LPS-stimulated neutrophils with extracts from Forsythia x intermedia flowers and leaves (25–100 μg/mL) resulted in a statistically significant reduction of IL-8 and TNF-α release (Figures 2A,B). The effect was especially significant in the case of TNF-α and for the leaf extract at the highest concentration of 100 μg/mL (40.4 ± 4.5% release of LPS-stimulated cells, p < 0.001). The flowers and leaf extracts at the highest concentration also decreased the surface expression of CD11a/ CD18 (p < 0.01 and p < 0.05, respectively). While the expression of CD11b/ CD18 was only effected by leaf extract at 100 μg/mL (p < 0.01; Figures 2C,D). The decrease in TNF-α production caused by the leaf extract was even more pronounced in monocytes/macrophage cells (p < 0.001 in all concentration tested; Table 2), whereas the activity of the flower extract was negligible (data not shown). The leaf extract, but not the flower extract, was able to significantly stimulate IL-10 receptor surface expression (p < 0.001 for all concentrations tested), as well as TGF-β production (p < 0.001 at 100 μg/mL) (Table 2).

**Table 2:** Effects of leaf extracts from *F. intermedia* and tested compounds on pro-inflammatory and anti-inflammatory functions of monocytes/macrophages.

	**TNF-α release [%]**	**Surface IL-10 receptor expression [%]**	**TGF-β release [pg/mL]**	**Death cells [%]^a^**
No stimulation	n.d.	100	n.d.	19.4 ± 1.8
Stimulation (+ LPS)	100 ± 1.4^#^	69.7 ± 1.4^#^	n.d.	21.3 ± 1.3
**STIMULATION + LEAVES EXTRACT**				
25 μg/mL	22.1 ± 0.4^***^	240.9 ± 9.9 ^***^	n.d.	21.3 ± 1.8
50 μg/mL	10.0 ± 1.0^***^	585.0 ± 57.3^***^	n.d.	21.8 ± 3.6
100 μg/mL	1.3 ± 0.1^***^	2564.0 ± 732.1^***^	128.2 ± 7.4^***^	21.8 ± 1.4
**STIMULATION + ARCTIGENIN**				
10 μM	16.9 ± 10.2^***^	64.7 ± 10.3	n.d.	19.8 ± 1.4
20 μM	11.4 ± 4.7^***^	57.0 ± 3.3	n.d.	22.0 ± 2.3
50 μM	10.1 ± 1.5^***^	63.4 ± 3.3	n.d.	23.2 ± 2.2
**STIMULATION + PHILLYGENIN**				
10 μM	82.0 ± 1.1^*^	1601.8 ± 460.3^***^	1551.1 ± 30.4^***^	23.8 ± 1.3
20 μM	21.5 ± 1.2^***^	2138.5 ± 568.9^***^	1741.7 ± 50.0^***^	25.6 ± 2.6
50 μM	8.4 ± 0.4^***^	3146.0 ± 678.1^***^	2202.8 ± 92.3^***^	21.7 ± 1.5
quercetin	2.1 ± 0.1^***^	64.9 ± 1.5	n.d.	21.9 ± 3.0

n.d., not detectable;

# P < 0.01 vs. not stimulated cells;

* P < 0.05 vs. stimulated cells;

*** P < 0.001 vs. stimulated cells;

a *compare with triton X-100- 100% of death cells*.

### Effect of *Forsythia x intermedia* flower and leaf extracts (FFE and FLE) on attachment of LPS-stimulated neutrophils to the TNFα-stimulated endothelium

**Figure 3:**
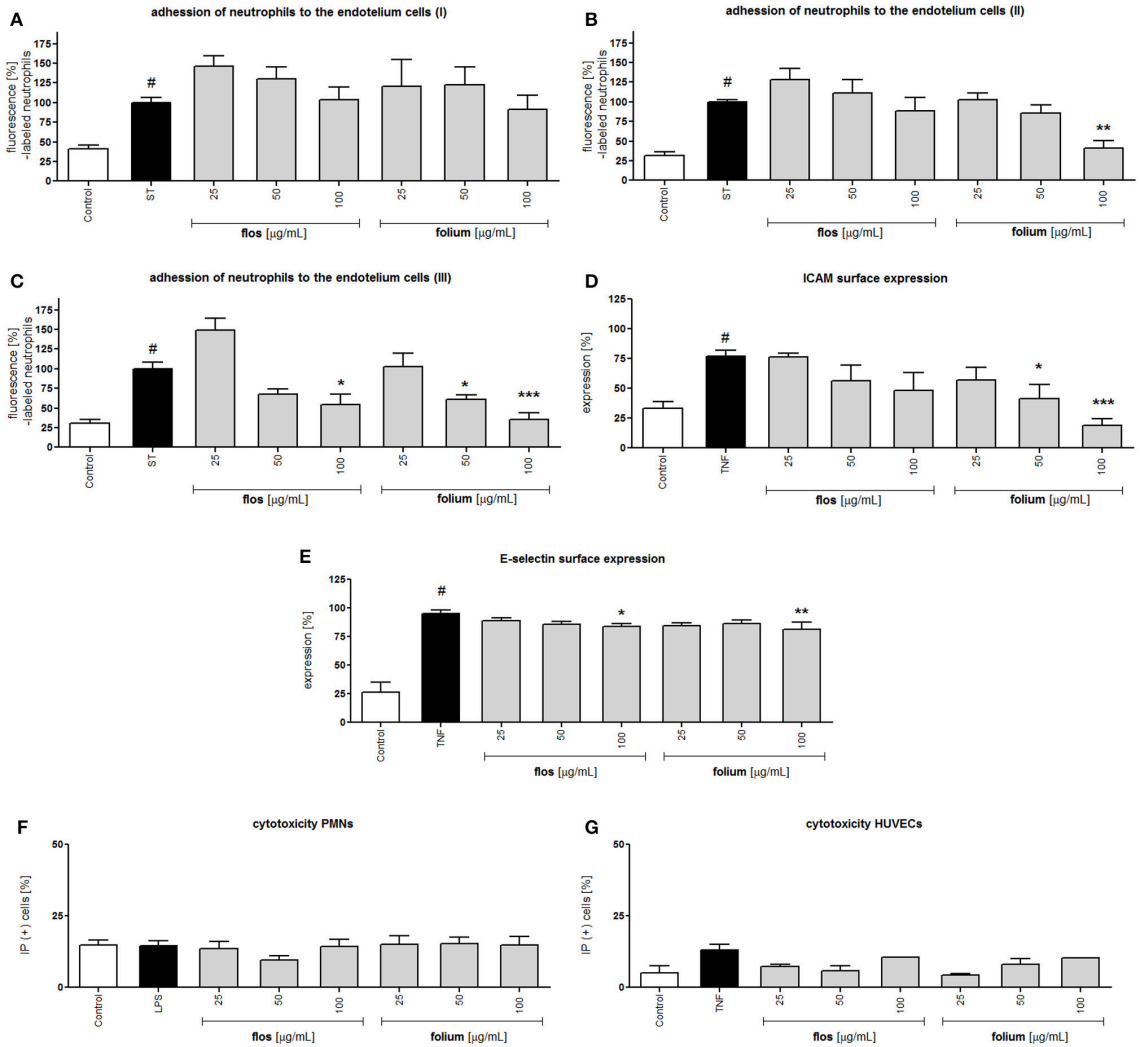
Effects of *F. x intermedia* flower and leaf extracts (FFE and FLE) on the attachment of neutrophils to endothelial cells: **(A)** neutrophils incubated with extracts in the concentration range 25–100 μg/mL; **(B)** endothelial cells incubated with extracts in the concentration range 25–100 μg/mL; **(C)** endothelial cells incubated with extracts in the concentration range 12.5–50 μg/mL and neutrophils incubated with extracts in the concentration range 12.5–50 μg/mL; **(D)** intercellular adhesion molecule-1 (ICAM-1) expression on the surface of stimulated endothelial cells [%]; **(E)** E-selectin expression on the surface of stimulated endothelial cells [%]; Percent of cells with diminished membrane integrity (propidium iodide positive cells IP (+)): **(F)** neutrophils; **(G)** HUVECs. Data are expressed as the mean ± SEM; at least three independent experiments were performed, and they were assayed in duplicate. Experiments were performed using cells from different donors. Statistical significance ^#^*P* < 0.01 compared to the non-stimulated control; *P < 0.05, **P < 0.01, ***P < 0.001 decrease compared to stimulated control (ST/ LPS/ TNF).

**Figure 4:**
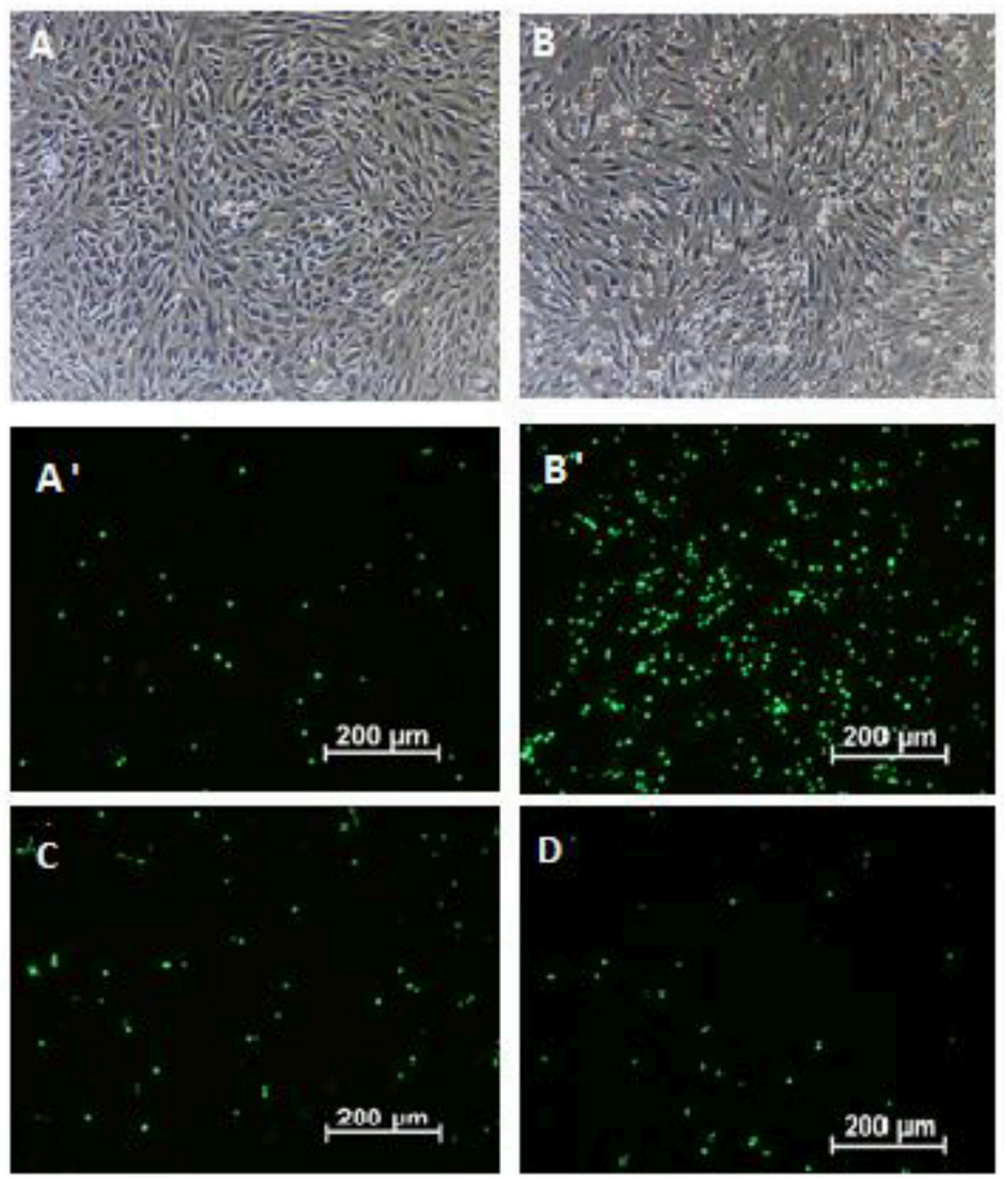
The attachment of calcein-AM load neutrophils to endothelial cells: non-stimulated neutrophils with TNF-α stimulated endothelial cells- visual light **(A)** and -fluorescence **(A**′**)**; LPS stimulated neutrophils with TNF-α stimulated endothelial cells- visual light **(B)** and -fluorescence **(B**′**)** LPS-stimulated neutrophils incubated with flower extract (50 μg/mL) and TNF-α-stimulated endothelial cells incubated with flower extract (50 μg/mL)-fluorescence **(C)** LPS-stimulated neutrophils incubated with leaf extract (50 μg/mL) and TNF-α-stimulated endothelial cells incubated with leaf extract (50 μg/mL)-fluorescence **(D)**.

Endothelial cells after stimulation with pro-inflammatory agents (e.g., TNF-α) are characterized by elevated expression of intercellular adhesion molecules (ICAMs), which interact with CD11a/CD18 and CD11b/CD18. The expression of E-selectin, which is responsible for the leucocytes rolling phase of leucocytes adhesion, was also induced. Although extracts showed an inhibitory effect on integrins surface expression at the highest concentration, the preincubation of neutrophils with both extracts did not prevent neutrophil attachment to endothelial cells (Figure 3A). The effect was more visible when HUVECs were preincubated with extracts. The leaf extract at a concentration of 100 μg/mL inhibited neutrophil attachment (*p* < 0.01), and this effect correlated with a significant reduction in ICAM surface expression (*p* < 0.05 and *p* < 0.001 at 50 and 100 μg/mL, respectively) but not with E-selectin (Figures 3B,D,E). Interestingly, the dual preincubation of neutrophils (12.5; 25; 50 μg/mL) and HUVECs (12.5; 25; 50 μg/mL) with extracts resulted in significant reductions in adherence both for the flower and leaf extracts (Figures 3C, 4). At the same time, the potential cytotoxic effect was also investigated by using a propidium iodide assay. At the concentrations tested, no significant reduction in membrane integrity of both cell types was observed in comparison to the controls (Figures 3F,G).

### Isolation of active compounds from leaf and flower extracts (FFE and FLE) and their effects on neutrophils and monocyte/macrophage function

The crude FFE and FLE extracts were fractionated using a Diaion HP-20 and eluted with a 4-step H_2_O-MeOH gradient (80: 20 → 0: 100) to obtain 4 main fractions (FF1–FF4 and FL1–FL4) based on their TLC and HPLC profiles. The activities of fractions were evaluated based on IL-8 inhibition and TNF-α release. Fractions FF1, FF3, and FL1–FL4 did not affect the IL-8 release at a concentration of 10 μg/mL. The incubation of neutrophils with fractions FF2 and FF4 resulted in a decrease of IL-8 production to 78.2 ± 2.5% and 48.4 ± 3.0%, respectively, relative to 100% production for the control cells. The results were in agreement with the fact that FFE caused a greater decrease in IL-8 production (Figure 2A). More significantly, the incubation of neutrophils with fractions FF4 and FL4 (10 μg/mL) resulted in a decrease of TNF-α production to 4.7 ± 0.2% and 34.5 ± 1.0%, respectively, relative to control cells. The following 11 compounds were isolated from the active fractions FF4 and FL4 using Sephadex LH-20 chromatography and preparative chromatography: forsythoside A, acteoside, (+)-epipinoresinol-4′-*O*-glucoside, (-)-matairesinol-4′-*O*-glucoside, (+)-phillygenin-4-*O*-glucoside, (-)-arctigenin-4′-*O*-glucoside, (+)-pinoresinol, (+)-epipinoresinol, (-)-matairesinol, (+)-phillygenin, and (-)-arctigenin. The structures of these compounds were confirmed by their ^1^H and ^13^C NMR spectra, which were compared with reference data (Rahman et al., 1990; Tokar and Klimek, 1998, 2004b). All tested compounds were not active at concentrations 10–50 μM; they did not reduce IL-8 release. Only the lignan aglycones (+)-pinoresinol, (+)-epipinoresinol, (-)-matairesinol, (+)-phillygenin, and (-)-arctigenin at concentrations 10–50 μM were able to significantly decrease the TNF-α release from stimulated neutrophils (**Figure 6A**), at a level comparable to the positive control quercetin (50 μM). These compounds also decreased TNF-α release from monocytes/macrophages (Table 2). However, in the monocyte/macrophage model, arctigenin has shown a more significant inhibitory effect (16.9 ± 10.2% TNF-α release from LPS-stimulated cells for arctigenin vs. 82.0 ± 1.1% from LPS-stimulated cells for phillygenin at a concentration of 10 μg/mL). Both compounds similarly decreased the IL-1β release from PMNs (*p* < 0.001 at all concentrations tested) (Figure 6B).

Additionally, phillygenin, but not arctigenin, was able to significantly stimulate IL-10 receptor surface expression (*p* < 0.001) and TGF-β production (*p* < 0.001; Table 2).

**Figure 5:**
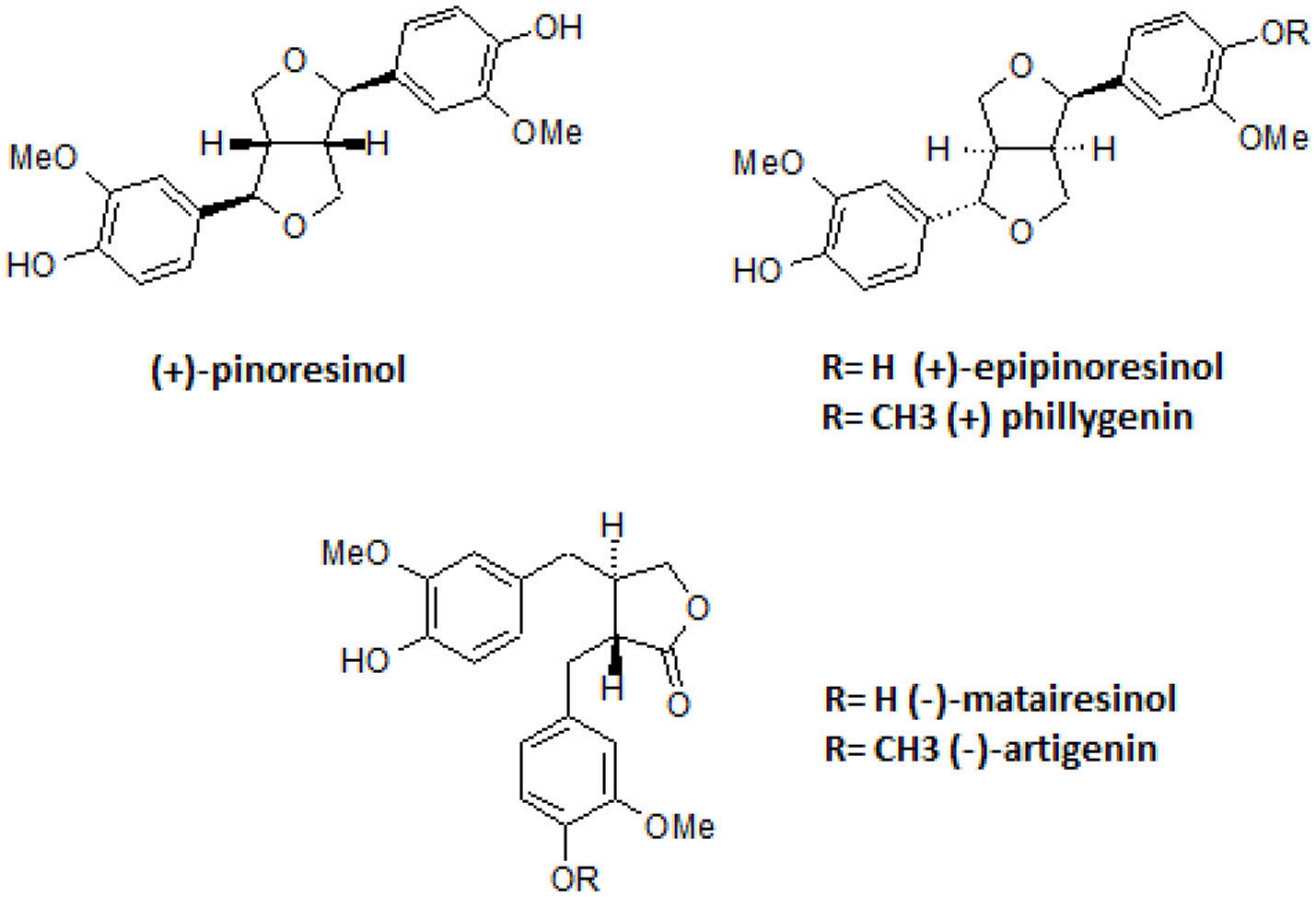
Structures of isolated lignans.

All compounds were not cytotoxic at the tested concentrations (Table S1, Table 2). The structures of the isolated active lignans are presented in Figure 5.

### Production of enterolactone from (+)-phillygenin-4-*O*-glucoside and (−)-arctigenin-4′-*O*-glucoside by human gut microbiota and its effect on cytokine/chemokine production and map kinase activation

*Ex viv*o incubation of human gut microbiota with phillyrin and arctiin for 24 h resulted in the production of enterolactone. The presence of enterolactone was confirmed by using a UHPLC-DAD-MS/MS method and comparing with standard compounds. The analysis of ethyl acetate extracts from human gut microbiota culture with both lignans revealed the presence of enterolactone with m/z 297 [M-H]^−^ and m/z 595 [2M-H]^−^, analyzed *via* ESI-MS in negative ion mode and with UV maxima of 217 and 274 nm (**Figure 8**).

**Figure 6:**
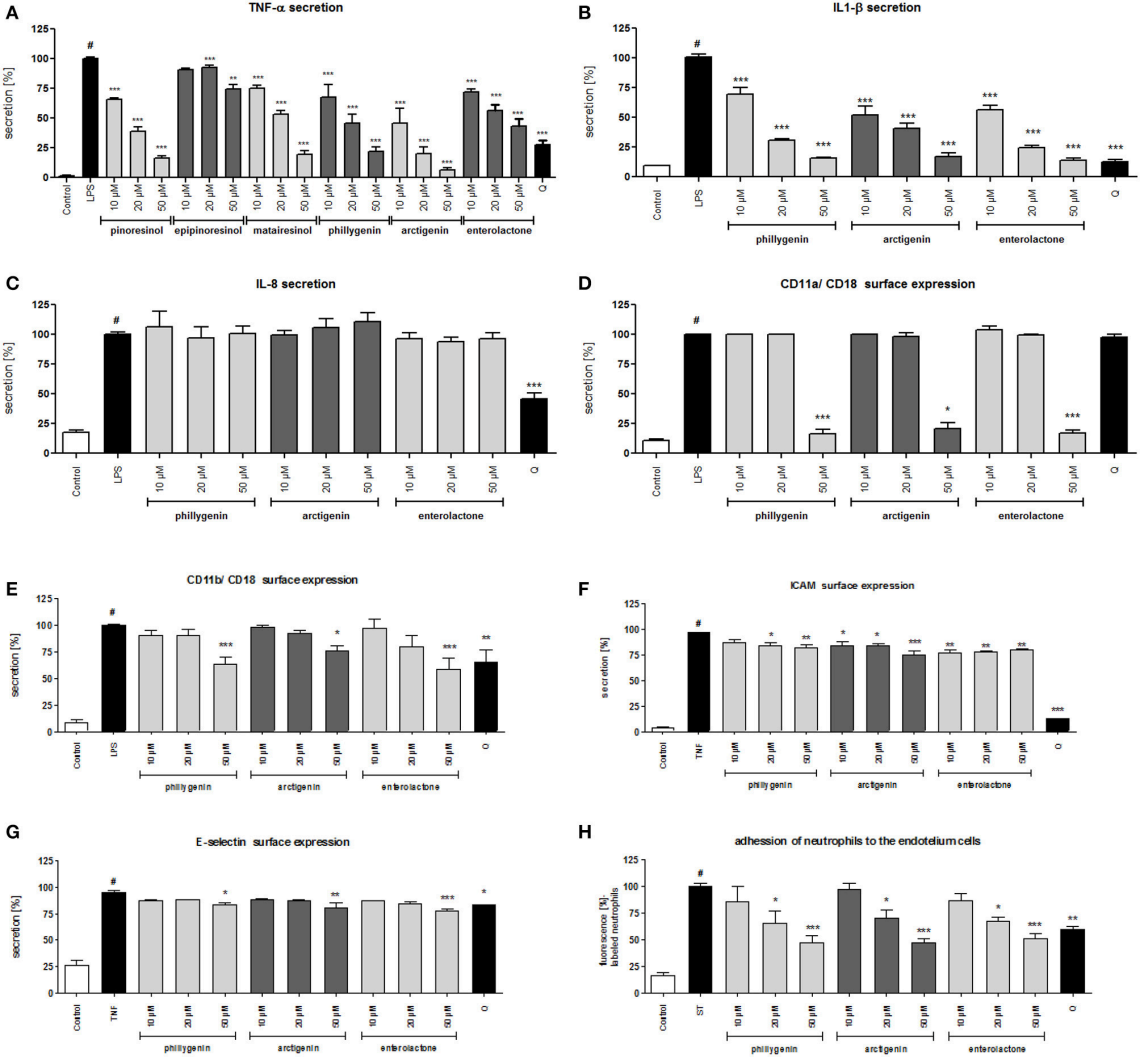
Effects of lignans at concentrations of 10–50 μM and quercetin (positive control) at a concentration 50 μM: **(A)** inhibition of TNF-α secretion from stimulated neutrophils [%]; **(B)** inhibition of IL-1β secretion from stimulated neutrophils [%]; **(C)** inhibition of IL-8 secretion from stimulated neutrophils [%]; **(D)** β2 integrin CD11a/CD18 expression on the surface of stimulated neutrophils [%]; **(E)** β2 integrin CD11b/CD18 expression on the surface of stimulated neutrophils [%]; **(F)** intercellular adhesion molecule-1 (ICAM-1) expression on the surface of stimulated endothelial cells [%]; **(G)** E-selectin expression on the surface of stimulated endothelial cells [%]; **(H)** the attachment of neutrophils incubated with compounds in the concentration range 5–25 μM to endothelial cells incubated with compounds in the concentration range 5–25 μM. Data are expressed as the mean ± SEM; at least three independent experiments were performed, and they were assayed in duplicate. Experiments were performed using cells from different donors. Statistical significance ^#^*P* < 0.01 compared to the non-stimulated control; *P < 0.05, **P < 0.01, ***P < 0.001 decrease compared to stimulated control (LPS/TNF/ST).

The biological activities of lignan metabolite was comparable to the activities of the lignan aglycones phillygenin and arctigenin (Figures 6A–C).

**Figure 7:**
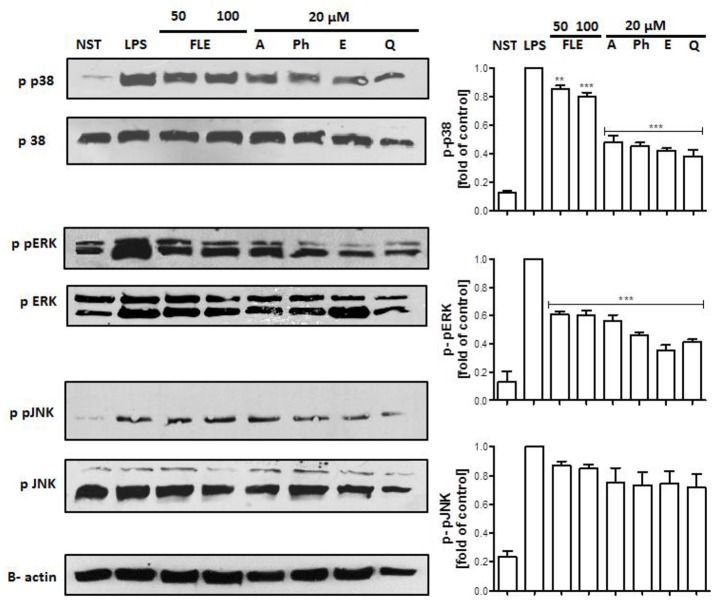
Effects of lignans at a concentration of 20 μM on the phosphorylation of p38 MAPK, p42/44 ERK, and JNK in LPS-activated human neutrophils. Phosphorylation of p38, p42/44 ERK, and JNK were analyzed by an immunoblot assay using antibodies against the phosphorylated form and based on total protein. Representative images from one of three experiments are shown. NST- not stimulated cells, LPS- stimulated cells, FLE 50- stimulated cells incubated with leaf extract at a concentration of 50 μg/mL, FLE 100- stimulated cells incubated with leaf extract at a concentration of 100 μg/mL, A- stimulated cells incubated with arctigenin, Ph- stimulated cells incubated with phillygenin, E- stimulated cells incubated with enterolactone, Q- stimulated cells incubated with quercetin. Statistical significance **P < 0.01, ***P < 0.001 decrease compared to the stimulated cells.

Lipopolysaccharide stimulation of human neutrophils resulted in the rapid phosphorylation of proteins, including p38 MAPK, p42/44 extracellular signal-regulated kinase (ERK), c-Jun NH2-terminal kinase (JNK), and transmigration of NF-κB-p65 from cytoplasm to the nucleus. Our data show that the LPS-induced phosphorylation of p38 MAPK and ERK1/2, but not JNK nor NF-κB-p65 transmigration, was decreased by all tested compounds at a concentration of 20 μM (Figure 7, Figure S1).

### Effects of phillygenin, arctigenin, and enterolactone on the attachment of LPS-stimulated neutrophils to the TNF-α-stimulated endothelium

All compounds were able to inhibit the LPS-stimulated surface expression of CD11a/CD18 and CD11b/CD18 at concentrations of 50 μM (Figures 6D,E). However, the effects of phillygenin and enterolactone were statistically more significant (*p* < 0.001 at 50 μM) than the effect of arctigenin (*p* < 0.05 at 50 μM) and were more significant or comparable to the activity of the positive control quercetin (50 μM). All tested compounds were able to slightly decrease the elevated expression of ICAM and E-selectin in TNF-α stimulated endothelial cells (Figures 6F,G). Those findings are in opposition to the activity of the leaf extract, which was mostly shown to prevent surface ICAM expression.

However, the dual preincubation of neutrophils (5; 10; 25 μM) and HUVECs (5; 10; 25 μM) with compounds showed a significant reduction of neutrophil adherence (Figure 6H).

## Discussion

*Forsythia* sp. fruit preparations are traditionally used to treat infectious and inflammatory diseases, especially in Chinese medicine, and the *in vitro* and *in vivo* activities of such preparations have been proven scientifically (Kim et al., 2000; Ko et al., 2006; Choi et al., 2007; Kuo et al., 2014; Zhang et al., 2016; Hwang et al., 2017). However, in temperate climates, such as in most European countries, fruits are not produced. The only viable sources of forsythia active compounds are the flowers and leaves. The aim of this study was to identify active compounds from chemically characterized forsythia extracts using human *in vitro* models of inflammation. As a positive control, we used the well-known natural product quercetin, which affects the pro-inflammatory functions of neutrophils and endothelial cells (Wei et al., 2001; Souto et al., 2011; Bhaskar et al., 2016).

*Forsythia suspensa* fruits predominantly contain phenylethanoids: (R)- and (S)-suspensasides, forsythoside A, suspensaside A; flavonoids: rutin; and lignans: pinoresinol-*O*-glucoside, epipinoresinol-*O*-glucoside, phillyrin, arctiin, and epipinoresinol (Guo et al., 2007; Cui et al., 2010). The fruits of other species (*F. koreana, F. viridissima*) contain mainly matairesinoside, arctiin, matairesinol, and arctigenin (Choi et al., 2003; Won et al., 2011). The same tendency in lignan composition is observed in the leaves and flowers: *F. suspensa*- pinoresinol-*O*-glucoside and phyllyrin; *F. viridissima*- matairesinoside and arctiin; *F. koreana* and *F. x intermedia*- pinoresinol-*O*-glucoside, matairesinoside, phyllyrin and arctiin. In the case of phenylethanoids, forsythoside A is present in *F. suspensa*, acteoside is present in *F. viridissima*, and both compounds are characteristic of *F. koreana* and *F. x intermedia*. The flavonoid rutin is characteristic of the whole genus (Kitagawa et al., 1988; Tokar, 2002; Tokar and Klimek, 2004b). Our study is in concordance with those observations, and we additionally emphasize the high biodiversity of secondary metabolites in *F*. x *intermedia* (Table 1, Figure 1). When we compared the chemical constituents of leaves and flowers of *Forsythia x intermedia*, the phytochemical profiles were similar. Only quantitative differences were observed. Rutin and arctiin are more abundant in the flower extract, whereas the leaves have higher levels of forsythoside A, acteoside, epipinoresinol glucoside, phillyrin, and phylligenin. In the neutrophil model, leaf extract was more biologically active, especially in decreasing TNF-α production in neutrophils and monocyte/macrophage cells and in increasing the anti-inflammatory function of monocytes/macrophages (increase in TGF-β release and IL-10 surface expression; Table 2, Figure 2). Additionally, the leaf extract attenuated the attachment of neutrophils to endothelial cells (Figures 3,4). The observed difference may be attributed to the high content of polysaccharides in forsythia flowers (Tokar, 2002), which may mask the extract bioactivity. Indeed, when both extracts were fractionated, the flower extract fraction FF4 and the leaf extract fraction FL4, which are rich in lignans (Figure 1B), showed similar activity in neutrophils (at a concentration of 10 μg/mL, TNF-α production decreased to 4.7 ± 0.2% and 34.5 ± 1.0%, respectively, relative to stimulated cells). Moreover, the flower extract fraction FF2 also showed a statistically significant decrease in IL-8 secretion (at a concentration of 50 μg/mL, production decreased to 21.2 ± 7% relative to stimulated cells). This may be attributable to a higher content in the flower extract (Figure 1A) and in fraction FF2, of rutin, a compound with known anti-inflammatory activity (Pastore et al., 2012). Stimulation of anti-inflammatory function in monocytes/macrophages was only observed for leaf extract and fraction FL4. The bio-guided fractionation of both active fractions led to the isolation of the following lignan aglycones: (+)-pinoresinol, (+)-epipinoresinol, (−)-matairesinol, (+)-phillygenin, and (−)-arctigenin (Figure 5). All of these compounds, in contrast to the glucoside forms (phillyrin and arctiin), inhibited the LPS-stimulated production of TNF-α both in neutrophils and monocytes/macrophages (Table 2, Figure 6A). The level of inhibition was comparable to the positive control quercetin. Interestingly, only phillygenin was able to stimulate the anti-inflammatory function of macrophages by inducing TGF-β release and IL-10 receptor surface expression (Table 2), which suggests that it has a role in the resolution of inflammation. However, arctigenin was inactive in this model. This observation is in some ways opposite to the observations of Hyam et al. (2013), who showed that arctigenin was able to polarize M1 macrophages to M2-like macrophages, which have an anti-inflammatory phenotype.

Interestingly neither phillygenin nor arctigenin influenced IL-8 release from LPS-stimulated neutrophils, whereas IL1-β secretion was statistically decreased (Figure 6B). The positive control, quercetin, was able to decrease the release of both cytokines (TNF-α, IL-1β) and a chemokine (IL-8), suggesting some differences in modes of action of lignans. Both lignans have shown some effects on neutrophil adhesion to endothelial cells (Figure 6F), which is an important step in neutrophil diapedesis to the site of inflammation. The stimulation of human neutrophils with LPS elicits a functional response through the activation of mitogen-activated protein kinases (MAPKs): p38 kinase, p42/44 extracellular signal-regulated kinase (ERK), and c-Jun NH_2_-terminal kinases (JNKs) (Nick et al., 1999; Arndt et al., 2004; Simard et al., 2015). The leaf extract mostly appeared to decrease p38 phosphorylation, whereas lignans, at a concentration of 20 μM, were able to attenuate the phosphorylation of both p38 and ERK kinases, but not JNK (Figure 7). The activation of p38 MAPK in neutrophils is connected with cell adhesion and the synthesis of TNF-α and IL-8 (Zu et al., 1998; Nick et al., 1999), and ERK activation also led to the expression of pro-inflammatory cytokines (Simard et al., 2015). However, we only observed a statistically significant effect on adhesion and effects on TNF-α and IL1-β release. One explanation for this is that there may be an additional effect of lignans on NF-*k*B inhibition, which has been shown to affect cytokine gene expression, although less markedly IL-8 gene expression (Cloutier et al., 2007). It also appears that, in human neutrophils, MAPK pathways affect cytokine production independently of NF- *k*B and *vice versa* (Cloutier et al., 2007). However, in our study none of tested lignan at concentration of 20 μM was able to attenuate the translocation of NF-κB-p65 from cytoplasm to the nucleus.

The observed significant inhibition of TNF-α and IL-1β production is of special interest for treating inflammatory disease. The pro-inflammatory effect of TNF-α mainly results from its capacity to stimulate the expression of adhesive molecules of endothelial cells and promote PMNs attachment to vascular endothelium, their degranulation and oxidative activity (Witko-Sarsat et al., 2000). Interleukin 1β, which is released from PMNs in concert with TNF-α promotes neutrophil recruitment, monocyte and neutrophils adhesion to vascular endothelial cells (Mitroulis et al., 2013).

Until now, lignan compounds have mostly been tested on mouse macrophage cells, not on human cells. This is important because murine models of innate immune response are difficult to translate to human pathological states (Zschaler et al., 2014). Nonetheless, arctigenin was shown, at a low concentration of 1 μM, to significantly inhibit the phosphorylation of the MAP kinases ERK1/2, p38, and JNK after LPS stimulation of the murine macrophage cell line RAW264.7 (Cho et al., 2004; Lee et al., 2010). As a result of this inhibition, the activation of transcription factor AP-1 was abolished, and the production of TNF-α was suppressed (Cho et al., 2004). Those results show a different mode of action of arctigenin and phylligenin in human neutrophils, where IL-1β, IL-8, and TNF-α expression seem not to depend on the JNK/AP-1 signaling pathway (Cloutier et al., 2003; Arndt et al., 2004).

At a higher concentration of 10 μM, arctigenin was also able to inhibit the phosphorylation of the PI3K/AKT pathway, and it was able to inhibit p65 in mouse peritoneal macrophages (Hyam et al., 2013). At concentrations of 1 and 10 μM, arctigenin was able to reduce the production of PGE_2_, and it inhibited COX-2 expression through attenuation of NF-*k*B (Lee et al., 2010). However, in our study the inhibition of cytokine release by arctigenin and phylligenin in human neutrophils model is rather not connected with the inhibition of NF-*k*B.

Phillygenin, in the same model, at concentrations of 10 and especially 100 μM, inhibited PGE_2_ formation (Lim et al., 2008), suggesting less pronounced anti-inflammatory activity than that of arctigenin. However, in our study, both compounds showed similar activity in a neutrophil model, and phylligenin appeared to be more specific in stimulating the anti-inflammatory function of human monocytes/macrophages.

The work of Lee et al. (2010) suggests that arctiin, the glycoside, is as active as arctigenin itself. This is in contrast with our study and the study of Wu et al. (2014). In the work of Lee et al. (2010), arctiin inhibited the production of pro-inflammatory cytokines (IL-1β, IL-6 and TNF-α) and PGE_2_, though relatively high concentrations of 25-100 μg/mL (~50-200 μM) were required. This may be due to the types of cells used (mouse or human) or deglycosylation during incubation (up to 48 h), which was not measured in the previous studies (Lee et al., 2010, 2011).

Lignan aglycones appear as active forms of glycosides present in plant extracts. The *in vivo* animal studies of phillyrin (10 and 20 mg/kg) and arctiin (30 and 60 mg/kg) showed significant anti-inflammatory effects (Wu et al., 2009; Zhong et al., 2013). This is supported by the fact that the glycoside forms of phillygenin and arctigenin are not well absorbed and/or not present in native forms in the blood and urine (Nose et al., 1993; Li et al., 2011; Wang et al., 2013, 2016). Additionally, the transformation of lignan glycosides, especially arctiin, by gut microbiota plays an important role in aglycone and enterolactone formation (Xie et al., 2003; Jin et al., 2007; Jin and Hattori, 2009).

**Figure 8:**
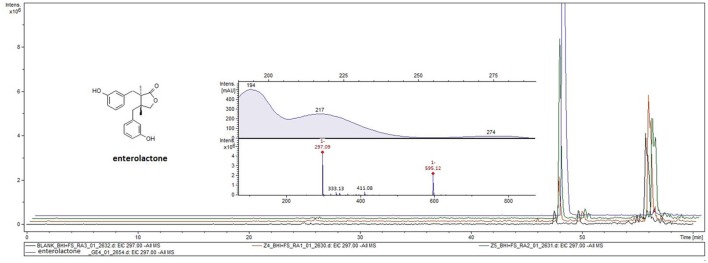
Enterolactone formation during incubation of (+)-phillygenin-4-*O*-glucoside (orange lane) and (−)-arctigenin-4′-*O*-glucoside (green line) with human gut microbiota in *ex vivo* culture. Enterolactone standard (blue line), blank culture (black line).

However, the anti-inflammatory activity of enterolactone has not been extensively investigated (Kivelä et al., 2008; Corsini et al., 2010; During et al., 2012). In our study, we confirmed the production of enterolactone by human gut microbiota from arctiin, and to a lesser extent from phillyrin (Figure 8), and we found that the activity of enterolactone is similar to that of primary aglycones (Figures 6A–H, Figure 7).

## Conclusions

The present study demonstrated that *Forsythia* x *intermedia* leaves and flowers are valuable sources of active lignans, which are attractive candidates for further research regarding their use in treating chronic inflammatory diseases that result from excessive activation of neutrophils. The observed decreases in the production of cytokines such as TNF-α and IL-1β depend on the inhibition of MAPK/ERK phosphorylation. The induction of IL-10 receptor expression and TGF-β release indicate that this group of natural products can provide leads for the development of novel therapies that modulate the inflammatory response.

## Author contributions

BM, AF, PC, MP, MW, BŻ-G, JP, and AKK performed the experiments. BM, AF, MO, and AKK carried out data analysis. AKK, BM, AF, and JP planned the experiments; AKK wrote the manuscript; AKK supervised all work. All authors revised and approved the final version of the manuscript.

### Conflict of interest statement

The authors declare that the research was conducted in the absence of any commercial or financial relationships that could be construed as a potential conflict of interest.
