# Supplementary material for: Lignans From Forsythia x Intermedia Leaves and Flowers Attenuate the Pro-inflammatory Function of Leukocytes and Their Interaction With Endothelial Cells
Source: Front Pharmacol. 2018 Apr 24;9:401. doi: 10.3389/fphar.2018.00401 (PMC5928392; doi:10.3389/fphar.2018.00401)
Supplement: Supplementary file 1 [file Data_Sheet_1.DOCX]

**SUPPORTING INFORMATION**

**Lignans from *Forsythia x intermedia* Leaves and Flowers Attenuate the Pro-inflammatory Function of Leukocytes and their Interaction with** **Endothelial Cells**

Barbara Michalak^a^, Agnieszka Filipek^a^, Piotr Chomicki^a^, Małgorzata Pyza^a^, Marta Woźniak^a^, Barbara Żyżyńska-Granica^b^, Jakub P. Piwowarski^a^, Agnieszka Kicel^c^, Monika A. Olszewska^c^, Anna K. Kiss ^a^*

^a^ Department of Pharmacognosy and Molecular Basis of Phytotherapy, Medical University of Warsaw, Banacha 1, 02-097, Warsaw, Poland

^b^ Department of Pharmacodynamics, Faculty of Pharmacy, Medical University of Warsaw, Centre for Preclinical Research and Technology (CePT), Banacha 1B, Warsaw, Poland

^c^ Department of Pharmacognosy, Faculty of Pharmacy, Medical University of Lodz, Muszynskiego 1, Lodz, Poland

* Corresponding author. Medical University of Warsaw, Banacha 1, 02-097 Warsaw, Poland. Tel./fax: +48 22 572 09 85

E-mail address: akiss@wum.edu.pl

**Table S1**. Influence of pure compounds on cells membrane integrity

**Cytotoxicity (propidium iodide staining positive cells [%])**

Neutrophils

|  | **10 µM** | **20 µM** | **50 µM** |
| --- | --- | --- | --- |
| **pinoresinol** | 17.4 ± 3.2 | 17.4 ± 3.8 | 14.3 ± 2.4 |
| **epipinoresinol** | 12.2 ± 0.6 | 10.2 ±0.1 | 13.7 ± 3.4 |
| **matairesinol** | 17.6 ± 3.5 | 17.3 ± 5.1 | 17.3 ± 3.7 |
| **phillygenin** | 8.4 ± 1.3 | 10.6 ± 3.1 | 21.1 ±4.0 |
| **arctigenin** | 20.1 ± 2.5 | 24.6 ± 1.7 | 22.6 ± 1.7 |
| **enterolacton** | 6.9 ± 2.3 | 6.4 ± 1.2 | 11.7 ± 4.4 |
| **quercetin** | - | - | 24.8 ± 3.2 |
| **control** | 18.7 ± 2.7 | | |
| **LPS** | 26.0 ± 2.4 | |  |

HUVECs

|  | **10 µM** | **20 µM** | **50 µM** |
| --- | --- | --- | --- |
| **phillygenin** | 7.0 ± 0.8 | 7.0 ± 0.3 | 10.1 ± 0.6 |
| **arctigenin** | 7.1 ± 0.5 | 10.3 ± 0.6 | 10.6 ± 0.3 |
| **enterolacton** | 7.3 ±0.7 | 6.9 ± 0.5 | 10.3 ± 0.3 |
| **quercetin** | - | - | 10.5 ± 0.4 |
| **control** | 4.9 ± 2.5 | | |
| **TNF** | 13.1 ± 2.0 | |  |

Results are presented as average ± SEM.

**Figure S1.** Image of pp38, ppERK, pp JNK and p65 each single blots.

**
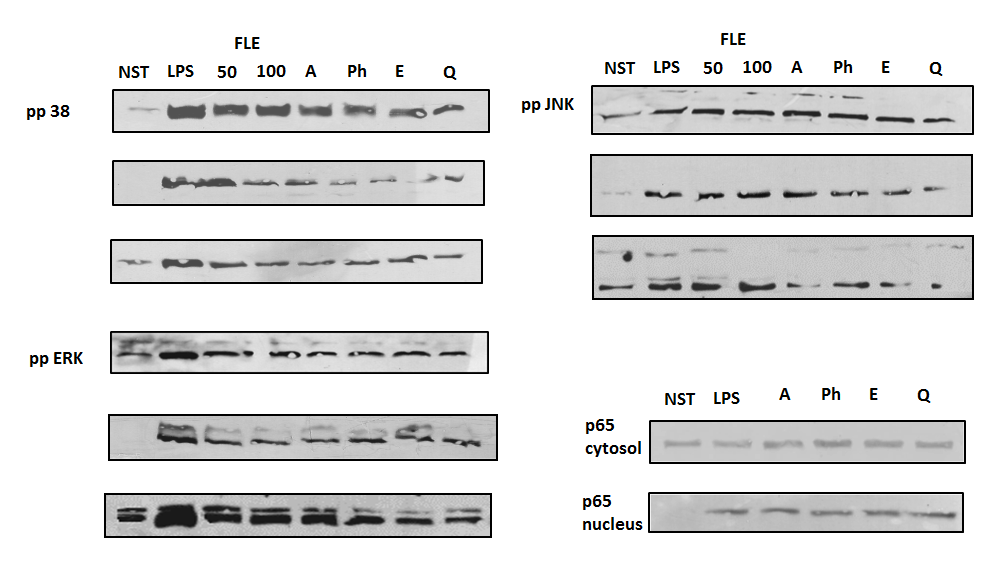
**
